# The Experiences of Fathers Who Have Offspring with Autism Spectrum Disorder

**DOI:** 10.1007/s10803-017-3035-2

**Published:** 2017-01-28

**Authors:** Alexander Burrell, Jonathan Ives, Gemma Unwin

**Affiliations:** 1 College of Medical and Dental Sciences, University of Birmingham, Birmingham, UK; 2 School of Social and Community Medicine, University of Bristol, Room G.4a, Canynge Hall, 39 Whatley Road, Bristol, BS8 2PS UK; 3 School of Psychology, University of Birmingham, Birmingham, UK

**Keywords:** Autism spectrum disorder, Qualitative, Fathers, Experiences, Acceptance

## Abstract

Research exploring parents’ experiences of having offspring with autism spectrum disorder (ASD) currently underrepresents fathers. This study aimed to develop an understanding of the experience of fathers, with a view to facilitating improved support. Eight fathers participated in semi-structured interviews exploring their experiences of fathering children with ASD. Fathers described their experiences as a path towards acceptance, with independence and integration for their offspring being key goals. Fathers saw themselves as advocates fighting obstructive services to access appropriate care. The value placed on formal and informal support varied, although the need for personalised support was emphasised. Enabling fathers to see their experiences as a journey, whilst engaging them on the important topics of independence and integration, may improve their experience.

## Introduction

Autism spectrum disorder (ASD) is a lifelong developmental disorder typically presenting in early childhood, characterised by impairments in social interaction and communication, and restricted, stereotypic patterns of behaviour (American Psychiatric Association 2013; World Health Organization 2004). Over recent decades, prevalence of ASD has increased globally including in Europe, the United States, China and Japan (Barnevik-Olsson et al. 2008; Honda et al. 2005; Kaye et al. 2001; Nevison 2014; Wong and Hui 2008). It is now estimated to affect 1% of the population in England (Brugha et al. 2011), whilst recent data from the United States Autism and Developmental Disabilities Monitoring Network observes a prevalence of 14.6 per 1000 in children aged 8 across their surveillance sites (Christensen et al. 2016). This change may relate to broadening of diagnostic criteria and increased social awareness of the disorder (Sadock et al. 2003). Regardless of the reason, increasing numbers of families are caring for offspring diagnosed with ASD, and may require support in doing so.

Parents’ experiences of diagnosis of ASD are known to be characterised by: feelings of grief and loss; fears concerning the long term impact of the diagnosis; a sense of loss for the life events their child would never experience; and, for some, the reaction is comparable to a death in the family (DeGrace 2004; Gray 2003; Hock et al. 2012). Caring for someone with ASD presents significant challenges: individuals with ASD may become distressed when routines are disturbed (DeGrace 2004), and have difficulties expressing their needs (Peppe et al. 2007). Medical comorbidities are also common, including a higher prevalence of epilepsy and sleep disorders when compared to the general population (Bauman 2010; Jokiranta et al. 2014). Furthermore, with the majority of adults with ASD remaining dependent on their families (Howlin et al. 2013, 2004), the role of a parent as a carer pervades throughout life. Both mothers and fathers of children with ASD show higher levels of parenting stress and psychological distress than parents of typically developing children (Baker-Ericzén et al. 2005; Davis and Carter 2008; Hastings et al. 2005), as well as a higher prevalence of both depression and anxiety (Almansour et al. 2013; Benjak et al. 2009; Kuusikko-Gauffin et al. 2013; Singer and Floyd 2006). These elevated levels of stress, and feelings of parental incompetence, can significantly reduce affective parental attachment to children with ASD, for both mothers and fathers (Goodman and Glenwick 2012). Children with ASD show markedly less secure attachment to their parents when compared to typically developing children (Rutgers et al. 2004), and parents’ subjective perceptions of their children’s attachment to them can contribute significantly to parental stress levels (Goodman and Glenwick 2012). The UK National Institute for Health and Care Excellence guidance on the management of ASD acknowledges these difficulties and their pervasion through parental experience, stating that parents of offspring with ASD should be offered regular personal, social and emotional support (National Institute for Health and Care Excellence 2013). As parents face the challenges of caring for children with ASD, an important emotional mediator is psychological acceptance. This process involves developing an awareness of, and embracing, difficult events without unnecessary and potentially distressing attempts to change their frequency or content (Hayes et al. 2006). Given the enduring nature of behavioural problems in people with ASD, traditional short term coping mechanisms, for example either avoidant or problem-focused strategies, may be unhelpful and indeed harmful over time for parents (Hastings et al. 2005). When comparing parental empowerment, a problem-focused coping method, to acceptance, Weiss et al. (2012) found that only psychological acceptance mediated the effects of child behavioural difficulties on parental mental health problems. Similarly, acceptance of ASD, and the creation of a ‘new normal’ within the family, has been reported qualitatively (DeGrace 2004; Luong et al. 2009). Quantitatively, increased acceptance of ASD has been found to mediate the relationship between challenging behaviour and parental wellbeing, reducing psychological distress (Jones et al. 2014).

A recent qualitative review exploring parents’ experiences of caring for a child with ASD developed a temporal model of parents’ experiences moving from the pre-diagnosis period to looking ahead to the future, drawing on the family life cycle model proposed by (Carter and McGoldrick 1988). The review found that parents face challenges including: feeling overwhelmed; developing a negative self-perception as a carer; feelings of guilt and blame; and receiving criticism in public (DePape and Lindsay 2015). This review identified six broad themes, which are:

*Prediagnosis* Associated with parent expectations of typical development, noticing atypical development, and searching for answers to explain what is perceived as atypical development. The stage of searching for answers is often a “prolonged and confusing process in which some parents received an incorrect diagnosis for their child” (p10).

*Diagnosis* Associated with “relief and devastation”, and “guilt and blame” (p10).

*Family life adjustment* Associated with, inter alia, managing challenging behaviour at home and in public; feeling judged by others; impact on family finances; feeling overwhelmed, stressed and exhausted; changes to spousal relationships (positive and negative); and impacts on relationships with other children.

*Navigating the system* Associated with attempts to access the best care and support they can, including challenges dealing with school systems and community services.

*Parental empowerment* Associated with attempts to take control through self-educating and developing coping strategies. The sub-theme ‘super-parents’ was developed, here, to describe the parent who, unsatisfied with service provision, becomes an expert and advocate for their child.

*Moving forward* Associated with accepting the diagnosis and looking towards the future. Parents may accept ‘the new normal’, and begin to identify the benefits of caring for an ASD child. ‘Looking towards the future’ involves parents seeking to prepare their ASD children for independence (i.e. living on their own or employment).

DePape and Lindsay note that not all the themes arose in all the 31 papers included in the review, and that more research is needed to confirm patterns of experience. They also note that in the included studies the maternal demographic is over-represented, with mothers constituting almost three quarters of respondents. They suggest this may be due to the desire of some studies to examine only the experiences of primary carers and, despite fathers increasingly adopting this role (Ellison et al. 2009), mothers do remain more likely to be primary carers. The impact of the father, even as secondary carer, is highlighted by some of the studies included in De-Pape & Lindsay’s (2015) review. Some studies report spouses growing closer as a result of jointly caring for their child (Aylaz et al. 2012; Hock et al. 2012; Markoulakis et al. 2012), whilst others reported gendered tension arising from mothers’ perception of not receiving enough spousal support (Gray 2003; Luong et al. 2009). For example, one mother participant in a 2003 study reported:

“Nine times out of ten it’s the mother who is [caring for the child]…because the father is working.…The father essentially has…respite care five days a week” (Gray 2003, p. 635).

A father in the same study told of his using work to avoid having to deal with a difficult situation at home:

“I was not working forty hours. Paid for forty hours a week, but I worked an average of sixty-five.…I think I did it to get away from [my daughter]” (Gray 2003, p. 635).

This is reminiscent of the findings of Genesoni and Tallandini, (2009) who, speaking in the context of the challenges fathers face in trying to reconcile their personal and work lives, noted that fathers will:

“frequently withdraw from this conflicting situation into their traditional role and do not attempt to change their habits; therefore they end up providing little support to their new families in terms of practical help.” (p316)

Even as ‘secondary carers’, then, fathers are still an integral and important part of a parenting dyad, whose experiences, actions and choices are both important in their own right and impactful on a range of other important outcomes.

A further limitation to existing research is that the fathers involved tend to be interviewed alongside mothers as a combined ‘parent’ experience (Altiere and von Kluge 2009; Farrugia 2009; Ludlow et al. 2012; Ryan and Salisbury 2012; Woodgate et al. 2008). Couple interviews, whilst useful in their own right, cannot always capture individual perspectives and needs, and do not always present individuals with the opportunity to raise matters that are of concern to only them and which they might not have shared with their partner. As such, a couple interview may only capture a negotiated or public front, rather than the personal perspective. Recent studies of fathers and fatherhood suggests that the experiences of fatherhood and motherhood can be fundamentally different, which might be explained by gendered embodiment and social and moral norms attached to gendered parenting (Doucet 2009; Ives 2014). This suggests that what we know about mothers in this context cannot uncritically be assumed to apply to fathers. Further, a recent study by Ives (2014) suggests that some fathers, cognisant of moral discourse around parenting, might not see their own worries and concerns as sufficiently important or legitimate to raise. It is therefore important that fathers are engaged with as fathers, and not just as one-half of a parenting dyad, so that their own, and possibly different, concerns and support needs can be considered.

With most of the current understanding of ASD parents’ experience coming from mothers, further exploration of fathers’ experience is needed and could improve the support offered to families by increasing awareness of father-specific issues amongst health and social care professionals. This study therefore aimed to explore the experience of being a father of someone with ASD in order to enable service providers to better understand the father perspective.

## Methods

### Design

Interpretive phenomenological analysis was used to investigate phenomena through the first-hand experience of participants—in this case the experience of being a father of someone with ASD. This approach explores the meaning people give to their lived experiences through an in-depth examination of a series of cases (Holloway 2005; van Manen 1990).

### Sampling and Recruitment

Purposive sampling was used to ensure participants had experience of the phenomenon in question. A sample size of eight to twelve participants was aimed for to ensure a sufficient quantity of data to adequately explore fathers’ experiences. Inclusion criteria were: self-identifies as male; self-identifies as a father of offspring (adult or child) with ASD; capacity to consent to participate. The only exclusion criteria was insufficient levels of English to complete an interview. No potential participants were excluded. Participants were recruited through two ASD support charities based in Birmingham and London, one using an online advert on their website and another sending a standardised recruitment email to their mailing list. After seeing recruitment material, interested individuals could contact the researcher and were then sent an information sheet.

This research received favourable ethical review from the University of Birmingham BMedSc Population Sciences and Humanities Internal Ethics Review Committee (reference number 2015–2016/C2/RA/18).

### Participants

Twenty fathers made initial contact with the researcher, and eight of these went on to participate. Twelve fathers chose not to participate following initial contact. Fathers who chose not to participate simply stopped replying, and did not provide a reason after follow up attempts. Participants’ age ranged from 45 to 56 with a mean age of 51 ± 3.5 years All participants were ‘White British’ except for one who was ‘White Other’. All participants had an undergraduate degree or higher level of education. Six participants were employed full-time, with one participant self-employed and one retired. The ages of offspring with ASD ranged from 8 to 24 with a mean age of 16.8 ± 4.79 years, and all offspring were male except one. All but one participant had other typically developing offspring, ranging in age from 8 to 24. For demographic information, see Table 1.

**Table 1:** Demographic characteristics of participants

Pseudonym	Age	Ethnicity	Marital status	Highest level of education	Employment status	Age of offspring with ASD	Gender of offspring with ASD	Age(s) of typically developing offspring
Robert	56	White British	Married	Undergraduate degree	Retired	20	Male	24
Richard	51	White British	Divorced	Undergraduate degree	Employed full time	19	Male	21
Chris	49	White British	Cohabiting with partner	Undergraduate degree	Employed full time	11	Male	8
Eric	53	White Other	Separated	Undergraduate degree	Employed full time	8	Male	20
Michael	54	White British	Married	Masters degree	Employed full time	24, 22, 20	Female, male, male	–
Steve	45	White British	Separated	Chartered accountant qualification	Self-employed	14	Male	16, 11
Tom	47	White British	Married	Undergraduate degree	Employed full time	15	Male	18
Joe	53	White British	Married	Undergraduate degree	Employed full time	15	Male	12

This sampling and recruitment strategy did lead to a sample that was self-selected, certainly not representative, and, unintentionally to a sample of men who had predominantly older children. Whilst this would raise a significant problem if our aim was to generalise, in this case we were not seeking a representative sample from which to generate generalisable and reproducible results; but rather seeking to generate insight into the experience of the men who did participate. Consistent with the goals, and limitations, of qualitative research, our sample allows us to gain insight into the issue under study from a particular perspective at a particular point in time, and provides material that can help us understand some aspects of the experience but not to comprehensively map it nor, for example, to draw meaningful comparison between sub-groups of participants. It does not, nor could it ever, allow us to make claims about all fathers with autistic offspring – and this is a feature of this kind of qualitative research generally.

### Data Collection

Data collection took place over a 6-week period from January to February 2016. Semi-structured interviews were used, which allowed participants to describe their experiences in their own words and style, enabling the collection of detailed, reflective first-person accounts consistent with interpretive phenomenological analysis (Holloway 2005; Smith 2009). Prior to beginning data collection, AB underwent a process of bracketing – recording biases, previous experiences of, or assumptions about, the phenomenon in question – in order to reduce the influence of preconceptions associated with the research (Tufford and Newman 2012). Throughout the data collection period, AB kept a study journal written immediately after each interview to reflect on both the emotive and practical elements of each interview (Koch 1998).

Interviews were broadly unstructured, with an initial open question being asked (“Tell me about your son/daughter with autism”) and conversation flowing from that, with topics being explored as they were raised by the participant. A topic guide was created, with input from ASD charities, to be used in case participants did not volunteer stories unsolicited. The topic guide included broad open exploratory questions on a number of topics, including: rewarding and challenging aspects of being a father of offspring with ASD; coping methods and support used; experiences with statutory and other services; perceptions of gender differences in being a parent of someone with ASD. Verbal and non-verbal prompts were used with care to avoid leading participants, and clarification was sought during interviews.

Prior to beginning the interview, participants filled in a consent form and demographic information form. Interviews were conducted in a number of locations, including the University of Birmingham and participant’s homes and places of work. All interviews were face-to-face, and lasted from 60 to 90 min.

### Data Analysis

Data analysis was concurrent with data collection. Interviews were transcribed verbatim, and reviewed repeatedly by AB in order to become familiar with the data, including identifying more detailed passages and meaningful statements (Smith 2009). Van Manen’s (1990) selective highlighting approach was then used in order to identify structures of the experience, sentences or sentence groups which appear thematic of the phenomenon. These were identified through a detailed line-by-line commentary of each individual transcript. Notes were then made to encapsulate these thematic statements, engaging in a hermeneutic conversation with each transcript as a set of parts and as a whole (Holloway 2005). These notes and structures were then condensed to produce emergent themes. These were then grouped into super-ordinate themes that encapsulated relationships between emergent themes. This process was conducted on each individual transcript. Notes made by AB were reviewed by JI and GU in a process of analyst triangulation, with disagreements in interpretation discussed and resolved to improve methodological rigor (Patton 1999). Moving from individual analysis to the group, relationships between super-ordinate themes from each transcript were then explored using mind maps, examining convergence and divergence between participants. This allowed the identification of essential themes, those which characterise the phenomenon across individual experience and are vital to the description and understanding of the lived experience.

## Results

Four essential themes were identified in the data: the path to acceptance; independence and integration; battlefield fathers; and heterogeneity of support. Each theme is described below, presented with selected illustrative quotations from participants.

### The Path to Acceptance

For these fathers, their experiences were described as a “path”, a journey with “milestones” along the way. This path began before the diagnosis of ASD, with all fathers experiencing feelings of frustration, guilt and embarrassment during the overall journey. This process, travelling along the path, was viewed retrospectively as a journey towards acceptance. It seemed that the ‘journey’ was a narrative participants used to make sense of their experiences, constructed on reflection. Once they had reached the point of acceptance they could look back and recognise the different stages as process – but they did not appear to have recognised themselves as being on a journey as they were experiencing it.

The journey, as it was narrated by these fathers, began before their children were diagnosed with ASD, and the start of the journey was the point at which they realised they had difficulties with their child’s behaviour. In the period leading up to diagnosis, however, fathers reported a gradual realisation that something was wrong, beginning with their child having difficulties and behaviours that they did not understand. These behaviours were often initially attributed to other conditions, commonly hearing and speech disorders, with ASD not considered: however as these problems persisted, participants reported considering more significant developmental problems; a realisation that “this isn’t right”. In most cases, it was someone outside the nuclear family who first mentioned ASD, including teachers and grandparents. This initiated contact with healthcare professionals, eventually leading to diagnosis. For Richard, who had found his son’s behaviour infuriating and perplexing, this diagnosis (when his son was around the age of ten) was the defining point of his path to acceptance after years of frustration:

“…it was described as…his wiring in his brain is twisted, it just does things a different way. And once we understood that, everything got a lot easier. And some of the things that would irritate you when you don’t understand…once you understood that is wasn’t him being…rude or obnoxious…it’s just [son]. And then that took a lot of pressure off…”

In contrast, participants whose children were diagnosed at an earlier age described diagnosis as a very difficult event, accompanied by an acute sense of loss. ASD was, at that time, seen as an external factor, a “monster” which had entered their life and led to the loss of the typically developing child they had expected and prepared for. Steve described his emotions:

“How did I feel? I mean obviously deeply upset, because as a father…you kind of want your son to do what you did which is play football and…have mates and do well at school and have a career. And you realise all that stuff isn’t gonna happen to your son…”

Some participants described feeling lost following the diagnosis, not quite knowing how to proceed. A first major step was seeking information, “reading up about it” in order to better understand their child and ASD. Seeking information about the disorder may help fathers to maintain some sense of control over their circumstances – where the diagnosis of ASD has ripped asunder their expectations of parenthood. Whilst this was reported as a vitally important step, allowing participants to begin to understand and explore the possible implications of the diagnosis, having a child with ASD still presented significant challenges which neither a diagnosis nor research could prepare them for: “the daily grind” as Eric described it. Practical difficulties with daily tasks, such as toileting or going to the shops, came to the fore; which could only really be understood through first-hand experience. Fathers often felt judged as parents and embarrassed in public when they were unable to control their children’s behaviour; struggling “to ignore people judging”, feeling like “a misfit” or “to blame” for their child’s challenging behaviour. Frustration was an oft-reported emotion, especially during conflicts at home over their child’s repetitive behaviours and inflexibility. Chris described reaching his limits:

“…I didn’t know I could be pushed to a limit of helplessness and aggression before all this happened… I’d be shouting and I don’t shout…all kids make parents shout but not to the point of frustration you get with somebody with autism, cause it’s just you can’t stand it…”

Frustration and anger were frequently followed by guilt, “the guilt after you’ve exploded”, with many fathers reporting feeling that their female partners seemed to manage their frustrations more effectively.

Whilst there was no specific event participants pointed to that led to acceptance of their child’s ASD and the associated challenges, all fathers described a change in their attitude to ASD over time, that they had “evolved” as a father. Michael described this talking about his three children with ASD:

“I think fundamentally, the change was that I began to accept and love them as they were, not as I wanted them to be or become…I ceased to think of it being a monster or an invasion of their life by an alien who had to be eradicated, and that it was them…it would always be them, it wasn’t a question of more effort by me to overcome it”

This kind of acceptance seemed to somewhat ameliorate the negative emotions and frustrations participants had previously felt, reducing frustration and conflict at home and at least partially alleviating embarrassment and the effects of perceived judgement in public. It seemed that frustration and anger arose from a combined desire to change their situation and a sense of helplessness. Realising they could not change their situation, but they could change their perception of it, seemed essential to many participants’ narratives of acceptance. Whilst this was reflected by all participants, it was also clear that despite having arrived at a state of acceptance and adaptation, the challenges of having a child with ASD were still significant and present. In this sense, ‘acceptance’ here does not refer to what might be termed ‘resolution of the diagnosis’ (Milshtein et al. 2010), but rather to a point at which a father ceases to view his experience purely in terms of frustration, guilt or anger and begins to view the child they have as a child *qua* child, as opposed to a child *qua* problem.

From this position, fathers were able to reflect on some of the positive changes that their experiences had brought about in them, including “being more tolerant…accepting and patient”. Eric described a change in his overall world view, brought about through his experiences, becoming less motivated by material gains and having a greater appreciation for “simple” things, including spending quality time with his son:

“…you come to appreciate the simple – the materia [sic] becomes less…important. And…let’s say not pack the time with…just ‘oh here’s a new toy, go and play with it’…I think that’s what I said with the journey, and learning curve…”

### Independence and Integration

Encouraging long term independence for their children with ASD, as well as imparting upon them the skills needed to integrate into wider society, was seen as important by most participants, with employment frequently raised as a significant objective.

Independence was seen as a key goal, and participants felt that it was their role to facilitate their child’s progression towards it. This often centred on teaching basic skills needed for an independent life, including cooking, cleaning and personal hygiene. Steve described this as a source of parental tension between mother and father with independence as his, but not his partner’s, main goal:

“…I said ‘look when he gets to eighteen, twenty, he shouldn’t be living at home, he should move somewhere where he can independent as an adult’, she just thinks he should be at home the whole time…her parents come round and they’ll brush his teeth and I’ll say ‘no no…he should brush his own teeth and we should supervise him’.”

Fathers often perceived employment to have a pivotal role in their children’s independence. This seemed to be because of the routine and structure it would provide, as well as providing them with a sense of achievement and success, as Joe describes:

“I’m not too bothered about whether it’s particularly well paid, I just hope it’s some sort of employment that he can do, and he’ll feel comfortable in. Doesn’t even have to be full time, just something to give him some purpose and a feeling of achievement, of success…”

Despite encouraging independence and employment, fathers often described themselves as managing expectations, both their children’s and their partner’s. This was a difficult balance to strike between encouraging their children to be positive but not unrealistic: a sense of “cautious optimism”, “trying to tell him the ways of the world”. There was a perception that succeeding in the world was harder for their children, leading to adjustment of goal-setting when compared to typically developing children. This generally seemed to be a positive experience, with fathers appreciating all achievements however small they may appear to other people.

Alongside independence, fathers seemed to express a desire for their children to be able to integrate and “contribute” to, as well as being accepted and appreciated by, society. This was both linked to societal acceptance of ASD making the father feel increasingly accepted and less judged or embarrassed, and a desire to see their children succeed with success generally defined in terms of independence, employment and integration.

Tom described the impact increased societal acceptance and awareness of ASD had on him:

“…I think there’s more of a level of acceptance, and that’s helped me…you don’t feel like you’re…a lone voice trying to…defend your child in a world that doesn’t care…”

As their children seemed to integrate more, and be more accepted, both through changes in societal attitude and through their children’s own experiences, fathers reported becoming better adjusted to having children with ASD. Chris expressed this clearly:

“…I think when he…his language got better, he started to partake more, he’s in mainstream school, he’s got friends, you see all of these things that you worried he wouldn’t have and you see…I dunno, everything changes really”

Some participants also discussed their children’s desire to integrate, and their subsequent efforts to teach them to “fit in”. They discussed their children’s desire to not be identified as different; especially difficult for those children who were in specialist education. For Joe, whose son was transitioning from specialist to mainstream education, this was especially important:

“…he doesn’t want the other kids in his class to know. So I’ve got this conversation to have with the college…’can you support him in the most discrete kind of way’. So I’ve been trying to…I’m forever saying to him ‘do this do that…if you want people not to know that you’re different don’t do that and don’t do that’…so I feel that’s my duty at the moment”

### Battlefield Fathers

Fathers frequently talked about themselves using military-style terminology, including “fight”, “battle” and “navigate”. This was most often referring to fighting “the system”, with the father as an advocate for his children with ASD, battling against a broken support system made up of barriers and bureaucracy.

All fathers talked about their role as an advocate; seeking out and accessing as much support as possible from both public and third sector services. Statutory support was often seen as incredibly difficult to “navigate”; with a telling comment from Steve that there was no “route map” or “welcome pack” to guide a father to sources of support. A lack of cohesion between services meant that accessing and obtaining each piece of support was a new battle to be fought. Support described included educational statements, disability living allowance and carers allowance, with the battle to access each service presenting a new challenge. Joe described this process as follows:

“You have to fight for everything, you have to reason with people who will…quote procedure at you, do their best not to help you in the politest way possible. And you have to take them on.”

This constant battle was a significant stressor, with Steve describing it as “hard work, very stressful, very draining, required a lot of research and very costly”.

When discussing this process of fighting, fathers often reflected on their role as an advocate and the skills needed for this role. The need for confidence, education and articulacy were mentioned, and fathers here considered themselves lucky to have those skills and expressed concern for those in a less fortunate position. As Robert put it:

“…I regard myself as quite articulate and…able to look after myself, but you just wonder how other people survive this sort of minefield, and that’s what worries me as well because some people just can’t cope with it…”

Despite possessing these skills, fathers often felt ignored by professional services when talking about their children. They frequently felt “misunderstood” or disregarded by professionals, as Tom described:

“…at times I think we felt…that…you weren’t being believed. ‘This is what’s going on’, but because that then didn’t fit in to any…other previous…research or ‘oh he’s doing this that means it’s caused by this’, we seemed to be in this vacuum at times…”

This was a difficult experience for fathers, who saw themselves as “experts” in regard to their own children and felt that professionals should give parents more of a voice in their children’s care, especially with a condition presenting across a spectrum. In Robert’s words:

“I think…that parents know their children and also parents um…every case is different so it’s difficult to say that, but…parents are…I think parents should be taken in to a lot more credence of what they say, and…take their advice and…listen.”

### Heterogeneity of Support

When discussing what support fathers accessed to assist their own coping, there was considerable heterogeneity. There was notable difference around what forms of support participants found useful, with different fathers relying on different mechanisms, both formal and informal, to aid their coping during challenging periods.

Formal support services aimed specifically at parents of children with ASD were discussed, run by third sector services based on a support group format. The majority of fathers seemed averse to this type of support for various reasons: for Chris, this support lacked a “personal” feel; Michael felt alienated by the “victim” mentality; Eric felt it was too “theoretical”; and Steve felt uncomfortable with “opening up” in that setting. Tom however, despite feeling that fathers were “afterthoughts” when support for parents was considered, still found father-specific support groups useful:

“…I found it very…helpful for me to go to that, to understand I wasn’t the only one that was going embarrassed, frustrated, impatient, angry then getting guilt tripped after being angry. A lot of people were all in the room…all seemed to be similar”

Even for Tom though, informal support from other fathers was preferred, with a social network formed through his son’s specialist school. Other participants felt no need for formal support, as spousal support was enough for them. This seemed to be related to the idea of parents as experts on their own children, with the mother and father supporting each other in meeting the unique challenges their children presented. The parents became their “own support network”, becoming more “self-reliant” as they became more experienced. Chris described parents providing each other with periods of respite as a “relay”, with the father stepping in when the mother had reached her limits and vice versa, with some participants seeing their main role being to spend time in the evenings, weekends and holidays doing activities with their children in order to provide respite to the mother. Tom described this when discussing his role:

“…it’s also to…share the burden. Burden, that’s a horrible word to use, but it is to do your bit. So when I’m around at weekends I very much see my role as his father to do things with him, to give my wife some respite. So I’ll take him up the park Sunday morning…then we’ll go for a walk up to the paper shop…things like that”

The value placed on peer support varied between fathers. Some fathers preferred to keep their friends separate from their difficulties at home, not discussing with friends the impact that the challenges of ASD were having on their lives. As described by Michael:

“I haven’t made it easy for my friends, because I have tended to become more introverted…it is a way of not wanting them to think that I am that tragic figure defined by autism. And so therefore not to mention it, on the surface to pretend that I have a relatively normal life.”

Others described how having a child with ASD had shaped their friendship groups, with those friends who were understanding and made an effort to get to know their children becoming closer friends. A similar process occurred with the extended family, with a distinct separation between those who were willing to help and those who were not, as Chris explained:

“…so you think you’ve got your family and your peers sorted by the time you’re thirty-five…and then this happens and there’s a real separation with people that can cope with it and people that can’t…and you start to eradicate the people that can’t cope with it cause they’re no use to you anymore.”

Relationships with peers who also had children with ASD or other developmental disorders were especially valuable for a number of fathers, allowing them to seek advice and honestly express their frustrations and emotions without fear of judgement.

## Discussion

Fathers in this study described similar experiences, and gave similar meaning to these experiences, as mothers of children with ASD. Moving towards acceptance of ASD, with a subsequent improvement in coping, has been expressed previously in research with mothers (Altiere and von Kluge 2009). This process has been compared to stages of grief (Kübler-Ross 2009), including in a recent study investigating the experiences of fathers in America (Frye 2015). Given the reflections of fathers in this study that diagnosis was comparable to the loss of a typically developing child, this seems an appropriate comparison. Arriving at this position of acceptance, for these fathers, involved moving past frustration and anger—which is a notable difference to the recorded experience of mothers, who tend to report sadness. Compared to mothers, fathers of offspring with ASD seem to report experiencing more anger than sadness, and attempt to suppress their negative emotions rather than express them (Gray 2003). Whilst this is consistent with research exploring gender and coping (Thoits 1995), hiding these frustrations from social networks or disengaging with professional support, as described by some fathers in this study, may be a barrier along the journey to acceptance, perhaps leading to a longer and harder journey than necessary. Encouraging fathers to express their frustrations and feelings of grief, whilst reassuring them that these are normal emotions to experience, may enable them to move towards acceptance and an increased resilience more easily. One way this might be achieved is through individual parental guidance meetings, allowing fathers to raise issues which are important to them and directly address them. However, this is unlikely to be a panacea, given the general reluctance of fathers to discuss these emotions with friends or family, and the reluctance of some to seek and accept professional support. Another option for fathers who are experiencing grief, frustration and anger may be simply to hear the stories of fathers who have survived the journey. Fathers in this study only processed their experiences as a journey, with a satisfactory end point, when reflecting back on them. To hear from other fathers that the challenging emotions they are experiencing are part of a process of moving towards acceptance, and recognising this earlier, may facilitate easier passage. The potential options discussed here may be encompassed by acceptance and commitment therapy (ACT; Hayes et al. 1999). As an approach focusing on parental acceptance of challenging negative emotions, overcoming negative thoughts, and identifying and moving towards personal goals, ACT has been shown to improve mental health outcomes of parents of offspring with ASD (Blackledge and Hayes 2006). Through accepting the futility of their efforts to change their situation and changing their perception of the challenges faced, as discussed by participants in this study, ACT may help fathers achieve psychological acceptance and reduce the perceived situational difficulties they experience, and should be considered as a support option when engaging with fathers—particularly those who are identified as struggling.

The perception of fathers in this study was of services being obstructive as opposed to facilitative. This is similarly expressed by mothers of offspring with ASD, with difficulties accessing services for their offspring frequently raised (Altiere and von Kluge 2009; Woodgate et al. 2008). The need to fight and navigate through “the system” was a source of stress for fathers in an already challenging situation, beginning with a lack of direction at diagnosis. This lack of information on available support was raised in a recent survey of parents in the United Kingdom, with the majority (61%) of parents dissatisfied with the level of post-diagnostic information provided (Crane et al. 2016). To better support both fathers and mothers at diagnosis, and to ensure they feel directed towards support, an electronic or paper resource containing information on regional services available for parents as well as for the individual diagnosed with ASD might be useful. However, as the father’s journey continues, and he becomes an expert in the needs of his own offspring, this level of general support may be less useful. To reduce the perception of service providers as a part of a system to battle against, health and social care professionals should ensure parents have been listened to when discussing the needs of their offspring.

Fathers in this study placed particular importance on their offspring becoming independent and integrating into wider society, and saw themselves as having an important role in helping their children achieve this. It may help fathers feel more engaged and positive about this role if professional services engage directly with these issues, acknowledging that this may be a concern, particularly for fathers: raising this topic and opening up space (either in a professional or an informal social setting) to talk about it may allow fathers to better express their needs and allow them to feel more supported in this role. Recent research with fathers transitioning to fatherhood has suggested, however, that simply opening up space to talk may not be enough, as fathers may be reluctant to raise concerns that they perceive as being ‘about them’ (Ives 2014). Rather, they may need active permission and invitation to talk about areas of concern to them. Additionally, given that being a father to someone with ASD is an ongoing experience with each developmental stage posing new challenges, this same principle will likely apply to all stages of the family life cycle (which may well be life-long). It would be important for such spaces to be available at all stages of the journey, until such point as they are no longer needed; from discussion about expectations of normality at the antenatal stage to discussion about employment and independence during adolescent education. These spaces will be useful for two discrete purposes: first as a way of supporting fathers who do not need anything quite as interventionist as ACT and, second, to create spaces where those who are struggling can both be identified and self-identify. What these spaces might look like, what form the support should come in, and who should provide it, are incredibly challenging questions; made more complex by the obvious and inevitable lack of resources and the difficulty of engaging with fathers who may not want it or recognise they might be in need. The data presented above show that different men appreciate different kinds of support, at different times, and some may have no interest in external support; so the one thing we can say with relative certainty is that one size will not fit all. Neither our data, nor any other data we have seen, provide a simple answer to this question; and we are wary of making a simplistic recommendation in response to these concerns that fathers need access to support groups or to dedicated expert professional services that are available throughout the life course etc. These would be no bad thing in principle, but the former seems unlikely to be accessed, and the latter is resource intensive to the point of being highly unlikely, if not impossible. We can, however, consider longer term approaches and theorise about what these spaces might need to look like and what changes might be necessary to allow them to be created. For this we draw on recent research that theorises ‘deliberative fatherhood’(Ives 2015), which proposes that in the absence of clear substantive success criteria for fatherhood, ‘good’ fatherhood has to be measured against formal process driven criteria based around the search for better practice; on the understanding that fatherhood is a moral and relational practice, that good practice is context dependent, and that fathering practice is fallible and uncertain. To quote at length:

“Conscientiously engaging in the process of deliberation with the goal of being a morally good father (and partner, and man), accepting that there will be reasonable disagreement about what achieving this goal involves, and being aware that achieving that goal requires negotiation and compromise, admits the possibility that he could get it ‘right’. It does not guarantee that he has. When we do not know precisely where the goal is, or what shape it is, the best we can do is ensure that we are running towards where we think it is, on the understanding that we might have to change direction. It is only by deliberating that we can ever become aware of the need to change direction. We do not have a clear and universal idea of what morally optimal fatherhood looks like, and until we do, a good father can only be someone who is actively trying to find out.” (p292-3)

This suggests that the way to support a father of an ASD child (or any father) is to facilitate the creation of spaces that allow this deliberation to occur; that does not judge or pretend that there is a clear and correct path to take along this journey; is responsive and available at the time it is needed; and accepts that different fathers and different families may have differently legitimate goals. These spaces might be literal physical spaces where discussions and deliberation can take place with others, or they may be metaphoric ‘spaces’ that allow, and give permission, for individuals to deliberate privately and in their own time. It seems unlikely that a single service or single provider could achieve this; nor that this could be achieved independently of cultural shifts in our thinking about fatherhood (and parenting) more generally; or without a move away from idealised notions of family and normalcy in general that is reliant on normatively loaded expertise that dictates in the abstract what is normal and what is acceptable. What is required, however, at a very basic level, is an incorporation of greater knowledge and understanding about ASD children and parents into current service provision, from antenatal care to education, that allows realistic expectations to be formed, conversations about ASD to be had, awareness to be raised, and support for parents to anticipate and reflect on problems and finding solutions that work for them. That anticipation and reflection will likely become more effective the more research is done in the area and the better we come to understand the range of experiences associated with parenting an ASD child.

Given the positive impact of wider public acceptance on fathers’ experiences, this study does support calls for further knowledge exchange between professionals and the general public about ASD (Pellicano et al. 2014). Increasing public awareness about the everyday realities of having offspring with ASD may reduce associative stigma (Farrugia 2009) in public, and allow fathers to feel that both they and their offspring are accepted by society, and therefore feel more able to accept ASD themselves.

Whilst fathers saw employment as an important goal for their offspring, they also expressed concern about how attainable that goal was. These concerns are well founded, with only 15% of adults with ASD in the United Kingdom in full-time paid employment (Rosenblatt 2008). Supported employment schemes, designed to assist people with disabilities find and retain employment, have been shown to be both cost effective and clinically effective. For example, the ‘Prospects’ scheme run by the National Autistic Society has achieved 67% employment rates (Howlin et al. 2005). Individuals with ASD who access these schemes may have improved self-esteem, reduced social isolation and improved access to social networks (Mavranezouli et al. 2013): seeing their offspring achieve these outcomes, or being aware of its possibility and how to help achieve it, would arguably be beneficial to fathers. Supported employment schemes are currently recommended by the National Institute for Health and Care Excellence for adults with ASD (National Institute for Health and Care Excellence 2012). Increasing awareness of these schemes amongst fathers may provide a greater sense of hope for the future, and allow fathers to assist in accessing these schemes when available.

### Limitations

This study has a number of limitations. As each participant completed a single interview, they could only reflect on how their experience had changed over time. A longitudinal study may better capture the evolution of fathers’ experiences. The sample in this study also lacked diversity in ethnicity, age, educational and employment status. As family background, including race/ethnicity, household income and education, has been shown to predict parents’ expectations for their offspring with ASD (Kirby 2016), variations in these demographic areas may lead to differences in experience. Fathers in this sample were recruited using methods involving ASD support charities, therefore participants are those who are at least aware of support services. Fathers who are not engaged with these services—and indeed those fathers who chose not to participate in this research—may have different experiences and opinions, and should be considered in future investigations. The homogenous nature of the participants in this research, and their active engagement both with support services and the research process, limits the theoretical generalisability of the findings presented.

The sample underrepresented fathers who have daughters with ASD, and it is unclear whether the findings are related to parenting a boy with ASD, or a child with ASD. It is possible that some fathers will have different expectations of, and attitudes towards, male and female children, and that may not have been captured in this study. Similarly, the age range of offspring with ASD in this sample is relatively narrow, with the majority of the sample in their teenage years or older. This relative lack of offspring in early childhood further limits the generalisability of this study: fathers with older offspring may perceive their situation through a different lens to that of fathers with younger offspring, who may have had a more recent diagnosis and would be at different developmental stages compared to the offspring in this sample. On the other hand, fatherhood studies in general do tend to focus on fathers with younger children, and in recognition that fathering continues beyond the early years this study provides some insight into fathering at a stage that is generally understudied.

## Conclusion

Fathers describe their experiences as a journey, with acceptance as a turning point at which their frustrations are reduced. Discussing this process with fathers as it is happening may allow a more positive perspective during difficult periods. Social acceptance and independence for their offspring are seen as important goals, which fathers’ journeys aim towards. By openly discussing these issues with fathers they may feel more engaged with service providers, and feel a greater sense of hope of achieving these goals. By ensuring fathers feel able to express their frustrations and feel heard by health and social care professionals, they may be able to move towards acceptance more easily.
